# K21 Compound, a Potent Antifungal Agent: Implications for the Treatment of Fluconazole-Resistant HIV-Associated *Candida* Species

**DOI:** 10.3389/fmicb.2019.01021

**Published:** 2019-05-24

**Authors:** Cathy N. John, Pedro M. D. S. Abrantes, Bhupesh K. Prusty, Dharam V. Ablashi, Charlene W. J. Africa

**Affiliations:** ^1^ Maternal Endogenous Infections Studies (MEnIS) Research Laboratories, Department of Medical Biosciences, University of the Western Cape, Bellville, South Africa; ^2^ Institute for Virology and Immunobiology, University of Wuerzburg, Wuerzburg, Germany; ^3^ HHV-6 Foundation, Santa Barbara, CA, United States

**Keywords:** HIV-associated candidiasis, antimicrobial compounds, *Candida*, fluconazole, antifungal susceptibility, broth microdilution

## Abstract

**Background/Objectives:** With mucocutaneous candidiasis being highly prevalent in HIV patients, the emergence of fluconazole-resistant *Candida* species forms a major challenge in treating and eradicating these infections. The objective of this study was to establish the antifungal activity of K21, a membrane-rupturing antimicrobial compound derived from a silica quaternary ammonium compound (SiQAC) with tetraethoxysilane (TEOS).

**Methods:** The study sample included 81 *Candida* species of which 9 were type strains and 72 were clinical isolates. Minimum inhibitory concentrations, synergy, fractional inhibitory concentration index (FICI), and time kill assays were determined by broth microdilution. Electron microscopy (EM) was used to determine the qualitative changes brought about after treatment with K21.

**Results:** K21 inhibited the growth of all fluconazole-resistant and susceptible *Candida* strains with only 2 h of exposure required to effectively kill 99.9% of the inoculum, and a definite synergistic effect was observed with a combination of K21 and fluconazole. EM demonstrated the presence of two forms of extracellular vesicles indicative of biofilm formation and cell lysis.

**Conclusion:** The study established the efficacy of K21 as an antifungal agent and with fluconazole-resistant candidiasis on the increase, the development of K21 can provide a promising alternative to combat acquired drug resistance.

## Introduction

The oral cavity may show the first signs and symptoms of HIV infection (Hirata, 2015), with the progression of HIV infection leading to frequent and recurrent episodes of highly severe oropharyngeal candidiasis (OPC) and systemic fungemia (Junqueira et al., 2012), often caused by fluconazole-resistant non-albicans *Candida* species (Mulu et al., 2013).

Currently, five main classes of antifungal agents exist, each with its own particular cellular target and mode of action (Liu et al., 2014). The triazoles (Fluconazole, Itraconazole, Voriconazole, Posaconazole and Isavuconazole) and allylamines (Naftifine and Terbinafine) inhibit synthesis of ergosterols by C14-demethylase and squalene epoxidase activity, respectively. The echinocandins (Caspofungin, Micafungin and Anidulafungin) interfere with cell wall synthesis. Damage to the cell wall results in disrupted membranes and as a result of osmotic fragility, cytoplasmic contents are spilled, thereby suppressing further fungal growth. The fluropyrimidine, 5-fluorocytosine, inhibits nucleic acid synthesis, interfering with normal cell metabolism, protein synthesis, a complete destruction of the cell interior and collapse of the cell wall (Kim et al., 2011).

The most commonly used antifungal agents in cases of HIV-associated OPC are azoles (Fluconazole, Itraconazole and Ketoconazole) and polyenes (Amphotericin B) (Patil et al., 2018). Although Fluconazole (FCZ) is reported to be the drug of choice (Sholapurkar et al., 2009), the improper and extensive use of the azoles has led to the emergence of fluconazole-resistant *Candida* and an increased occurrence of non-*albicans Candida* species, causing refractory mucosal candidiasis in HIV patients with advanced immunosuppression (Witaningrum et al., 2018), resulting in increased morbidity and mortality (Sardi et al., 2013; Africa and Abrantes, 2017). Non-*albicans Candida* species such as *Candida glabrata* are reported to show a higher rate of FCZ resistance (11–13%) followed by *Candida tropicalis* (4–9%), and *Candida parapsilosis* (2–6%) (Cleveland et al., 2012; Pfaller et al., 2015). Being fungistatic rather than fungicidal, FCZ has been implicated in both inherent and acquired resistance (Berkow and Lockhart, 2017).

The Centres for Disease Control and Prevention (CDC, 2014) revealed an alarming 7% increase in fluconazole-resistant *Candida* species, creating the need for the development of new antifungals which demonstrate both an increase in drug potency and improved tolerability, particularly in patients who are frequently affected by resistant *Candida* species. The toxicity of the polyenes, the inadequate oral absorption of the echinocandins, combined with the fungistatic activity and resistance to azoles, necessitates a change in treatment options.

Quaternary ammonium compounds (QAC) are nitrogen containing compounds with a N (nitrogen) atom attached to four different covalent bonds (Jiao et al., 2017). They are highly potent in combating antimicrobial resistance and the critical need for the development of new and effective antimicrobial drugs incorporating QAC is imperative in the fight against resistant species (Jennings et al., 2015; Jiao et al., 2017). K21 is an antimicrobial compound developed by Dr. Kirk Kimmerling in 2011 (Ablashi, 2015). The process of hydrolysis and condensation of a silica quaternary ammonium compound (SiQAC) with tetraethoxysilane (TEOS) resulted in the production of a 3-dimensional antimicrobial macromolecule with multiple arms of membrane rupturing potential (Prusty et al., 2014). Depending on the form of the SiQAC starting material, either methanol (if methacryloxy), or ethanol (if ethacryloxy) SiQAC, is generated as a by-product. The ethacryloxy SiQAC-derived K21 is preferred for materials being placed in the oral cavity and has been reported to exhibit an inhibitory effect on the growth of *Porphyromonas gingivalis*, *Escherichia coli, Streptococcus mutans, Actinomyces naeslundii* and *Enterococcus faecalis* (Gong et al., 2012a,b, 2014) along with the development of K21-coated sutures which may offer the potential of reducing post-operative infection and bacteremia (Meghil et al., 2015). The positive charges on K21 act by drawing the negatively charged microbe toward the material, while the long 18 carbon chain tails pierce the cell walls causing lysis. The use of K21 as an antiviral against HSV-1, HHV-6A, and HHV-7 has also been reported (Gulve et al., 2016), but to our knowledge, its antifungal activity has not been investigated.

The present study aimed to evaluate K21 as an effective antifungal agent against resistant *Candida* species by establishing the time required by the compound to inhibit the growth of *Candida* species and demonstrating synergy between K21 and FCZ.

## Materials and Methods

### *Candida* Isolates Used in This Study

*Candida* type strains were obtained from the American Type Culture Collections (ATCC, Manassas, VA) and National Collection of Pathogenic Fungi (NCPF, Public Health Culture Collections, England) and included *Candida albicans* (ATCC 90028 and NCPF 3281), *Candida krusei* (ATCC 2159), *C. glabrata (*ATCC 26512), *Candida dubliniensis* (NCPF 3949a), *C. tropicalis* (ATCC 950), *C. parapsilosis* (ATCC 22019), *Candida lusitaniae (*ATCC 34449), and *Candida kefyr* (ATCC 4135). These served as quality controls in the K21 antifungal susceptibility assays and checkerboard synergy and time kill assays.

Once the activity of K21 on the type strains was established, its activity was further tested on a selection of fluconazole-resistant (*n* = 62) and -susceptible (*n* = 10) HIV-associated oral *Candida* species previously isolated (Abrantes et al., 2014) and stored with consent at −80°C in Pro-Lab Microbank microbial preservation vials (Cat. no. PL.170/M, Pro-Lab, Canada). This was an *in vitro* study of stored species for which ethical clearance was granted by the Biomedical Research Committee of the University of the Western Cape. The stored isolates were revived by growth in 10 ml Sabouraud dextrose broth (Cat. no. CMO147, Oxoid, UK) and incubation at 37°C for 3–5 days. Purity of growth was confirmed by streaking broth cultures onto solid Sabouraud dextrose agar (Cat. no. 84088, Sigma-Aldrich, USA) and incubating at 37°C for 24–48 h.

### Preparation of K21

A solution of K21 in 50% ethanol was obtained from KHG fiteBac Technology (USA). Stock solutions were prepared by dissolving the compound in acetone and then in sterile distilled water, with resulting concentrations ranging from 0.98 to 249.9 μg/ml.

### Antifungal Susceptibility Assays

Minimum inhibitory concentration values of FCZ against nine *Candida* strains were determined by broth microdilution according to the Clinical and Laboratory Standards Institute M27A3 approved standard protocol (Clinical and Laboratory Standards Institute, 2008). Fresh colonies of *Candida* from Sabouraud dextrose agar were suspended in sterile saline solution (0.85% NaCl) and the cell density adjusted to a 0.5 McFarland standard, yielding a yeast stock suspension of 1.5 × 10^6^ CFU/ml. The working suspension was prepared by making a 1:50 dilution of the stock suspension followed by a 1:20 dilution with Roswell Park Memorial Institute (RPMI) 1640 medium (Cat. no. R6504, Sigma-Aldrich, USA) buffered with MOPS (N-Morpholino-propanesulfonic acid) 0.165 M adjusted to pH 7.0, resulting in an inoculum concentration of 1 × 10^3^–5 × 10^3^ CFU/ml. The FCZ (Cat. no. 8929, Sigma-Aldrich, USA) drug concentration ranged from 0.12–256 μg/ml with sensitivity to FCZ indicated by CLSI approved breakpoint MIC values of ≤8 μg/ml, intermediate or dose-dependent susceptibility MIC between 16 and 32 μg/ml and resistance MIC ≥64 μg/ml (Eraso et al., 2008).

Disk diffusion assays were not performed in the present study due to the inability of K21 to diffuse into the agar media.

The microdilution test for K21 was performed in 96-well microtiter plates containing 100 μl of 2-fold serial dilutions of K21 in 100 μl RPMI medium. The plates were then inoculated with 100 μl of the inoculum and incubated for 24 h at 37^°^C. The MIC value readings were recorded both visually and spectrophotometrically using an Anthos 2010 spectrophotometer (Biochrom, UK) at 450 nm (Schwalbe et al., 2007; Wong et al., 2014). MICs were defined as the lowest concentration of K21 inhibiting visible growth or 100% of the microorganism. Based on CLSI criteria, 100% growth inhibition was observed as clear wells in the plates. The positive control included 100 μl of 25 μg/ml of FCZ in 50 μl inoculum, while 100 μl inoculum without K21 served as the negative control. Antimicrobial activity was confirmed by the lack of growth after transferring 10 μl of the sample from the clear wells onto Sabouraud dextrose agar and incubating at 37°C for 24 h.

The readings were also interpreted by adding 40 μl iodonitrotetrazolium chloride (INT) dye (Cat. no. 18377, Sigma-Aldrich, USA) into the wells and incubating for a further 2 h. The MIC values were read by the change in color. The concentration range of K21 was 0.98–249.9 μg/ml.

### Antimicrobial Synergism

Synergism between K21 and FCZ was determined using the nine type strains and the CLSI M27A2 and CLSI M27A3 microdilution checkerboard techniques (Clinical and Laboratory Standards Institute, 2002; Clinical and Laboratory Standards Institute, 2008). A starting inoculum was prepared by inoculating a tube of saline with fresh colonies of *Candida* and adjusting to a 0.5 McFarland standard (1.5 × 10^6^ CFU/ml of yeasts). A 1:10 dilution was done by adding 1 ml aliquot of inoculum to 9 ml RPMI yielding a working inoculum of 1.5 × 10^5^ CFU/ml. Broth microdilutions involving 2-fold serial dilutions were performed with K21 alone and FCZ alone and double concentrations of K21 and FCZ in combination so that serial dilutions contained various concentrations of the combinations of K21 and FCZ. The concentration range of K21 was 0.49–249.9 μg/ml and for FCZ, it was 0.12–64 μg/ml. A positive growth control containing inoculum without K21 and FCZ was included for each isolate and the negative control consisted of RPMI alone. The plates were incubated at 37°C for 24 h and inhibition of growth was confirmed by recording the turbidity and by the addition of 40 μl INT dye. The MIC values for K21, FCZ and the K21/FCZ combinations were observed.

To assess the interactions of the drug combination, the data obtained by visual reading were further analyzed using the fractional inhibitory concentration index (FICI) using the formula FICI = FICA + FICB where:

$$\mathrm{FICA}\;=\;\frac{\mathrm{MIC}\;\mathrm{of}\;\mathrm{drug}\;A\;\mathrm{in}\;\mathrm{combination}}{\mathrm{MIC}\;\mathrm{of}\;\mathrm{drug}\;A\;\mathrm{alone}}$$

$$\mathrm{FICB}\;=\;\frac{\mathrm{MIC}\;\mathrm{of}\;\mathrm{drug}\;B\;\mathrm{in}\;\mathrm{combination}}{\mathrm{MIC}\;\mathrm{of}\;\mathrm{drug}\;B\;\mathrm{alone}}$$

FICI values were interpreted as follows: FICI ≤0.5 = Synergy; >0.5 to ≤4 = Indifference; >4 = Antagonism (Johnson et al., 2004).

### Time-Kill Assay

A time-kill assay was conducted to assess the rate of fungicidal activity of K21 against FCZ-susceptible *C. albicans* (ATCC 90028) and FCZ-resistant *C. glabrata* (ATCC 26512). The study was performed by a method previously described and evaluated by Klepser et al. (1998). Broth microdilution was performed with a starting inoculum of 1–5 × 10^5^ CFU/ml and K21 was tested at concentrations equal to MIC, ½ MIC, and ¼ MIC obtained by the microdilution checkerboard method. Plates were incubated at 37°C for 24 h prior to removing a sample for the determination of colony counts at predetermined time points of 0, 2, 4, 6, 8, 12, and 24 h. A 40 μl sample was streaked onto sterile SDA plates. Colony counts were performed using a Gallenkamp 20/CX-300 colony counter (Gallenkamp Co. Ltd., UK). K21 was considered to have fungicidal activity when there was a reduction in microbial growth of ≥3log_10_ decrease in colony count after 24 h, resulting in about 99.9% reduction in CFU/ml relative to the initial inoculum. Fungistatic activity was considered as a reduction in growth lower than 99.9% or < 3log_10_ in CFU/ml from the initial inoculum after 24 h (Locke et al., 2018).

In addition, *C. albicans* (ATCC 90028 and NCPF 3281), *C. glabrata* (ATCC 26512) and *C. dubliniensis* (NCPF 3949a) were tested for synergism by the time kill study. The microtiter plates were challenged with K21 and fluconazole alone and with K21 and fluconazole in a 1:1 combination at their final concentrations of ½ MIC and ¼ MIC.

“Synergy” was defined as ≥2 log reduction in colony count for a given combination compared to the colony count obtained with the most active single agent and “Antagonism” was defined as ≥2 log increase in colony count for a given combination compared to the colony count obtained with the most active single agent (Zusman et al., 2013), while a >2 log and <2 log log increase or decrease in colony count at 24 h with the combination compared with that of the most active single agent alone was reported as “indifference” (Johnson et al., 2004).

## Electron Microscopy (EM)

### Scanning Electron Microscopy (SEM)

A volume of 200 μl of samples from microdilution wells of K21 (½ MIC 31.24 μl), FCZ (½ MIC 8 μl), and K21 + FCZ (½ MIC 15.62 + 4 μl, respectively) were prepared for EM after treatment with K21 at 2, 4, 6, and 24 h. Untreated *C. albicans* served as the control.

The cells were harvested by centrifugation at 5,000 rpm for 10 min to obtain a pellet. After discarding the supernatant, the pellets were washed three times for 5 min each with 0.1 M phosphate buffered saline (PBS) pH 7.4, fixed with 2.5% glutaraldehyde (Cat. no. G5882, Sigma-Aldrich, USA), washed thrice in PBS for 30 min, followed by two washes of 5 min each in distilled water. Cells were dehydrated in graded ethanols (50, 70, 90, and 100%), followed by critical point drying, sputter coated with gold–palladium for 60 s (Quorum Q15OTES system) and observed in a Zeiss Auriga field emission scanning electron microscope at 5 kV.

### Transmission Electron Microscopy (TEM)

Samples from ½ MIC wells treated with K21 were harvested by centrifugation at 5,000 rpm for 10 min to obtain the fungal pellet. After discarding the supernatant, pellets were washed with 0.1 M PBS (pH 7.4) three times for 5 min each, the supernatant was discarded and pellets were fixed in Karnovsky solution (4 ml 10% formaldehyde +0.2 ml of 25% glutaraldehyde) in 1 ml 10x PBS, followed by washing first in PBS twice for 5 min each, then in distilled water before discarding the final supernatant. Cells were then fixed in 4% low melting agarose solution and immediately transferred to 4°C or ice for 30 min to solidify the agarose. The solidified pellets were cut into small sections and washed in PBS for 5 min. After 2 washes in distilled water, cells were dehydrated in graded ethanols (30, 50, 70, 90 and 95%) twice for 10 min each in 100% ethanol for 5 min each, followed by embedding in a mixture of LR White (Cat. no. L9774, Sigma-Aldrich, USA) and ethanol in proportions of 50:50, 25:75 and finally, 100% LR White. Ultrathin sections (80 nm) were collected on nickel grids and stained with 2% uranyl acetate (AGR1260A, Agar Scientific, UK) and lead citrate for 5 min each. Grids were examined using a Tecnai 20 transmission electron microscope operating at 200 kV at magnification between 8,000× and 34,000×.

### Statistical Analyses

Data was analyzed using SPSS software programme (SPSS Version 24; SPSS Inc, Chicago, IL, USA) and were expressed as the mean (standard deviation) of at least three independent experiments. Student’s paired *t*-test was employed to compare K21 and FCZ results. *p* ≤ 0.05 indicated significance.

## Results

### Comparison of the Antimicrobial Activity of FCZ and K21 on Their Own and in Combination

**Figure 1:**
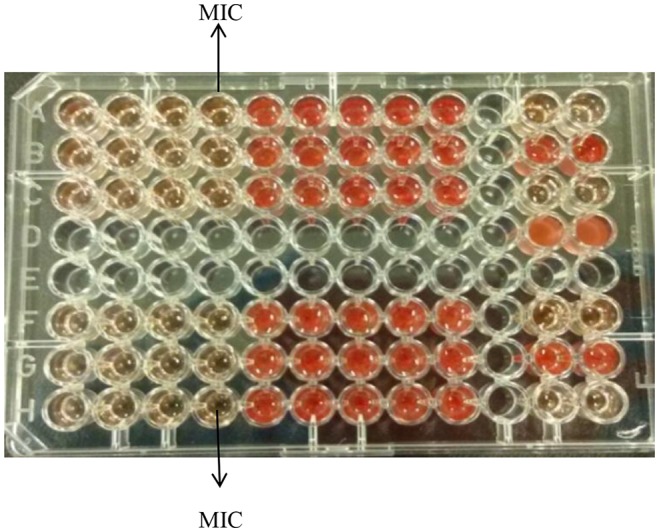
Broth microdilution assay for K21. Media control shows no growth (Row A, wells 11 and 12); Positive control show growth (Row B, wells 11 and 12); Negative control shows no growth (Row C, wells 11 and 12); Solvent control shows growth (Row D, wells 11 and 12).

Figure 1 demonstrates the broth microdilution test in a 96 well microtiter plate. The clear wells in the plate indicate the minimum inhibitory concentration (MIC) of K21. The presence of viable cell growth appeared turbid and was enhanced by the addition of INT dye.

Table 1 shows the susceptibility of the nine type strains. When treated with FCZ alone, *C. albicans* (ATCC 90028 and NCPF 3281) showed MIC values of 16 and 32 μg/ml, respectively, while *C. krusei* (ATCC 2159) and *C. glabrata* (ATCC 26512) both showed an MIC value of 64 μg/ml. The MIC values for *C. dubliniensis* (NCPF 3949a) and *C. tropicalis* (ATCC 950) were 2 and 1 μg/ml, respectively, while the MIC for *C. parapsilosis* (ATCC 22019) was 2 μg/ml and *C. lusitaniae* (ATCC 34449) and *C. kefyr* (ATCC 4135) were both 1 μg/ml.

**Table 1:** Microdilution checkerboard method to determine K21/FCZ synergy using *Candida* type strains.

Species	Individual MICs		Combination MICs		ΣFICI	Interpretation
			FIC A	FIC B		
*Candida*	K21 MIC (μg/ml)	FCZ MIC (μg/ml)	K21 MIC (μg/ml)	FCZ MIC (μg/ml)		
*C. albicans* ATCC 90028	62.48	16	31.24	8	1	Indifference
*C. albicans* NCPF 3281	62.48	32	62.48	16	1.5	Indifference
*C. krusei* ATCC 2159	62.48	64	62.48	16	1.25	Indifference
*C. glabrata* ATCC 26512	62.48	64	62.48	16	1.25	Indifference
*C. dubliniensis* NCPF 3949a	62.48	2	1.95	0.5	0.28	Synergy
*C. tropicalis* ATCC 950	62.48	1	0.97	0.25	0.26	Synergy
*C. parapsilosis* ATCC 22019	62.48	2	3.9	1	0.56	Indifference
*C. lusitaniae* ATCC 34449	124.95	1	0.97	0.25	0.25	Synergy
*C. kefyr* ATCC 4135	124.95	1	1.95	0.5	0.51	Indifference

≤0.5 = synergy, >0.5 to ≤4 = indifference (Johnson et al., 2004).

For K21, all the type strains, except *C. lusitaniae* (ATCC 34449) and *C. kefyr* (ATCC 4135) exhibited a MIC of 62.48 μg/ml.

Table 1 shows that K21 enhanced the effect of FCZ, while in the last five species listed, there was a major drop in MIC when K21 and FCZ were combined, compared to their individual MICs. A synergistic effect was observed in *C. dubliniensis* (NCPF 3949a), *C. tropicalis* (ATCC 950) and *C. lusitaniae* (ATCC 34449). Indifference was observed with *C. albicans* (ATCC 90028 and NCPF 3281), *C. parapsilosis* (ATCC 22019), C. *kefyr* (ATCC 4135), *C. glabrata* (ATCC 26512), and *C. krusei* (ATCC 2159). The MIC values of K21 alone showed higher concentrations compared to those of FCZ. There was no antagonistic effect found in the study.

**Figure 2:**
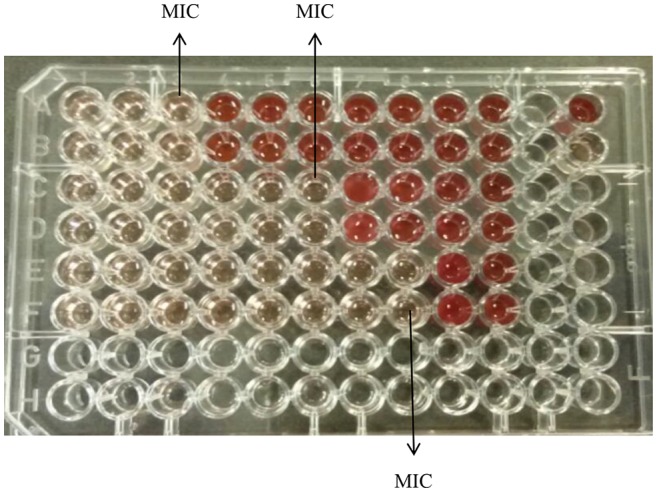
Checkerboard broth microdilution assay for K21 and FCZ synergy of *C. dubliniensis* (NCPF 3949a). K21 (Rows A and B); Fluconazole (Rows C and D); Combination of K21 and FCZ (Rows E and F); Positive control (Row A, well 12); Negative control (Row B, well 12).

Figure 2 demonstrates synergistic activity of K21 and FCZ on *C. dubliniensis* (NCPF 3949a) in the checkerboard microdilution assay.

### Time-Kill Assays of K21 on Its Own and in Combination With FCZ

The strains that exhibited synergy and indifference with the FICI indices were subjected to the time kill study, using ½ MIC and ¼ MIC combinations.

**Figure 3:**
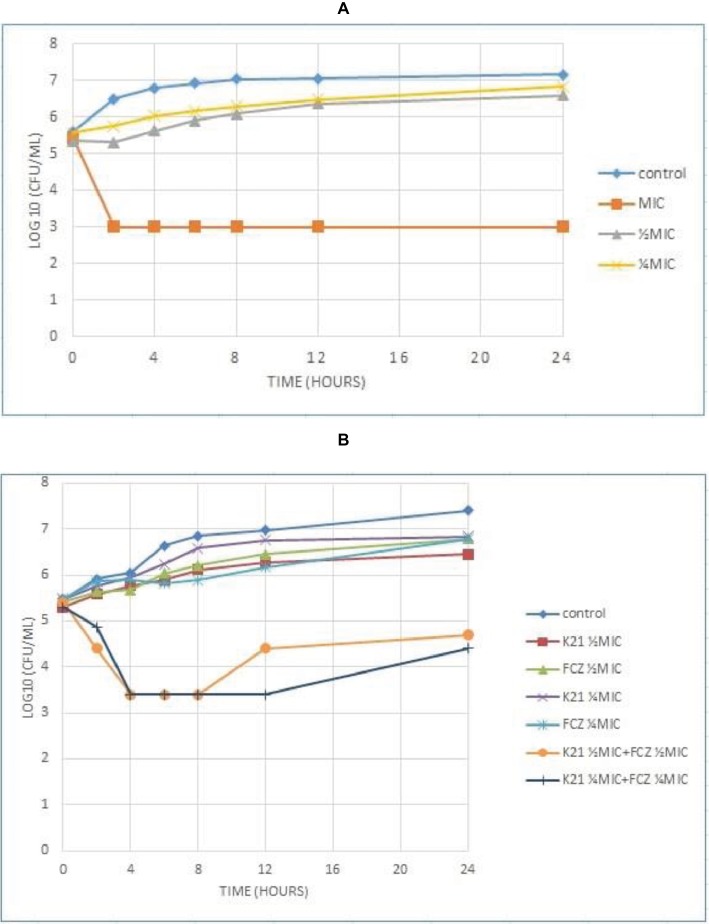
Time kill curve showing the log_10_ of CFU/ml versus time of FCZ-susceptible *C. albicans* (ATCC 90028) when exposed to various concentrations of K21 alone **(A)** and K21 + FCZ **(B)**.

The time required by K21 to inhibit the growth of *Candida* was determined and since no growth occurred at MIC, *C. albicans* (ATCC 90028) was used as a susceptible control, along with ½ MIC and ¼ MIC concentrations of K21. Figure 3A shows the time kill curve plotted against *C. albicans* vs. different concentrations of K21. Analysis of the graph showed a steady growth curve for *C. albicans* reaching a plateau at around 10 h. The MIC concentration showed a reduction in fungal growth at 2 h (≥3log_10_ of CFU/ml or lower than 99.9% reduction in microbial growth of the initial inoculum) which continued for 24 h. The ½ MIC showed a rapid growth curve up to 6 h with slower growth continuing up to 24 h. A similar curve was obtained for ¼ MIC.

Both K21 and FCZ tested at concentrations of ½ MIC and ¼ MIC combinations showed a reduction of *C. albicans* (ATCC 90028) from 4 h till 8 h (Figure 3B). There was a 2log_10_ of CFU/ml reduction in the growth in ½ MIC combinations of K21 and FCZ compared with killing by the most active single agent. At ¼ MIC combinations, a 2.5log_10_ difference was observed compared to both K21 and FCZ alone.

**Figure 4:**
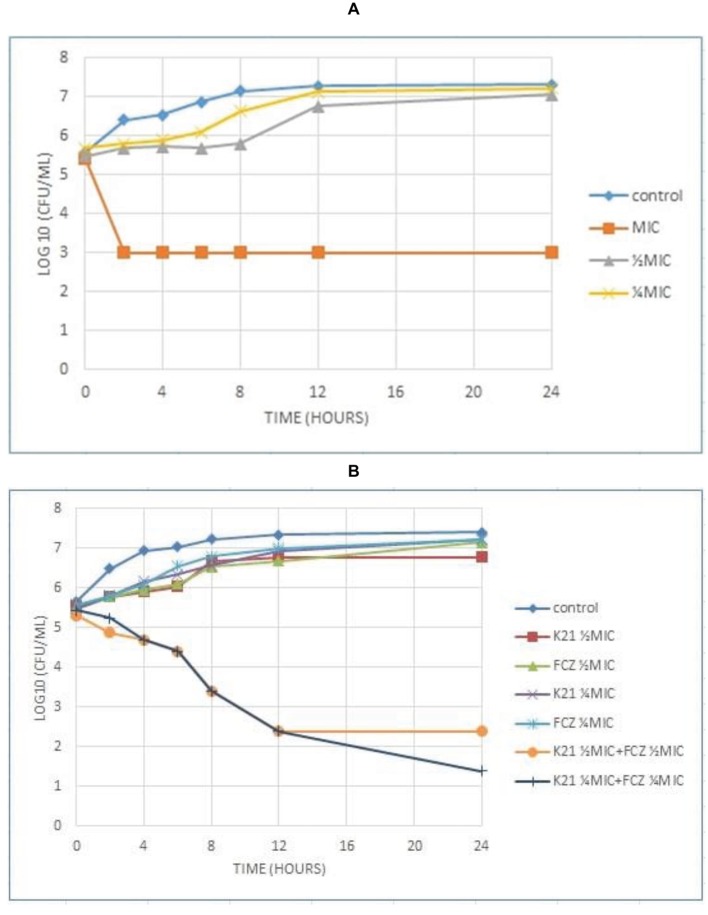
Time kill curve showing the log_10_ of CFU/ml versus time of FCZ-resistant *C. glabrata* (ATCC 26512) when exposed to various concentrations of K21 **(A)** and K21 + FCZ **(B)**.

Analysis of the graph (Figure 4A) shows a steady growth curve for the FCZ-negative control *C. glabrata* up to 6 h after which it plateaued. The ½ MIC and ¼ MIC presented a growth peak up to 6 h of exposure showing a fungistatic activity with a reduction in microbial growth of ≤3log_10_ of CFU/ml or lower than 99.9% of the initial inoculum. At 2 h, the MIC showed fungicidal activity with a reduction ≥3log_10_ of CFU/ml or about 99.9% reduction in microbial growth relative to the initial inoculum. In both the *Candida* strains tested, no viable cells were detected after 24 h indicating the fungicidal effect of K21.

Figure 4B demonstrates the time kill graph plotted against FCZ-resistant *C. glabrata* (ATCC 26512). There was a steady reduction in the growth of the cells till 12 h. A 4.5log_10_ difference at ½ MIC combinations was shown when compared to the single agent and a 5.5log_10_ difference to the starting inoculum. However, at ¼ MIC combinations, a < 2log_10_ reduction in the cell growth was observed. There was no synergy exhibited at ¼ MIC combinations of K21 and FCZ.

**Figure 5:**
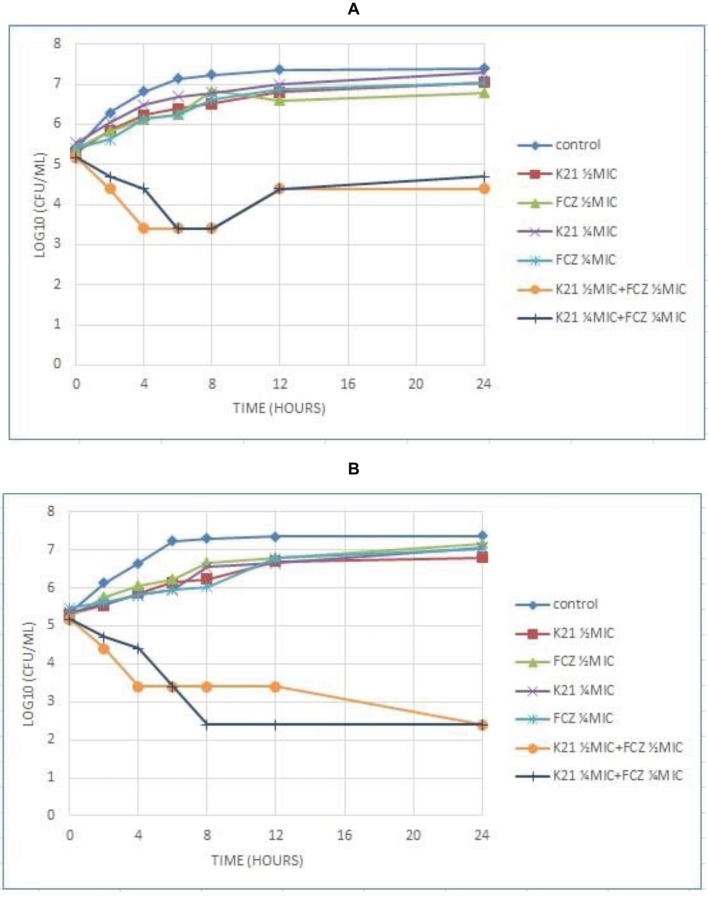
Time kill curve results of K21 and FCZ against **(A)** *C. albicans* (NCPF 3281) and **(B)** *C. dubliniensis* (NCPF 3949a).

Figure 5A shows *C. albicans* (NCPF 3281) at ¼ MIC concentrations of K21 and FCZ combinations with a reduction of cell growth seen at 8 h and an increase in cell growth at 12 h, continuing till 24 h. At ½ MIC combinations, growth reduced at 4 h with growth increasing from 12 to 24 h. There was an approximate 2.8log_10_ difference and 2.5log_10_ difference in the viable cell counts at ½ MIC and ¼ MIC combinations respectively, compared with the cells treated with either K21 or FCZ alone.

*C. dubliniensis* (NCPF 3949a) exhibited a 4.5log_10_ difference at both ½ MIC and ¼ MIC combinations of the drugs to their single drug concentration (Figure 5B). At ½ MIC combinations, there was a reduction in the viable count at 4 h, which remained constant till 12 h and reduced at 24 h. A reduction in viable cells was observed at ¼ MIC combinations at 8 h, remaining so at 12 and 24 h.

The antimicrobial effect of K21 was then tested against HIV-associated *Candida* isolates, of which 91.9% were *C. albicans*, 6.5% were *C. glabrata,* and 1.6% were *C. dubliniensis.* FCZ results showed that among the FCZ-susceptible strains (Table 2), a MIC range of 0.12–1 μg/ml was observed for *C. albicans* with a MIC_50_ of 0.5 μg/ml. The MIC range for *C. dubliniensis* was 0.03–0.5 μg/ml with a MIC_50_ of 0.5 μg/ml demonstrated for both *C. dubliniensis* and *C. glabrata.* On treatment with K21, the MIC ranged between 31.24 and 62.48 μg/ml for *C. albicans* with MIC_50_ values of 62.48 μg/ml recorded for *C. albicans* and *C. dubliniensis* and 31.24 μg/ml recorded for *C. glabrata.*

**Table 2:** Effect of K21 on HIV-associated *Candida* clinical strains.

*Candida n* (%)	Fluconazole (FCZ)			K21		
	Mean MIC (*SD*)	MIC range (μg/ml)	MIC_50_ (μg/ml)	Mean MIC (*SD*)	MIC range (μg/ml)	MIC_50_ (μg/ml)
**FCZ-susceptible**						
*C. albicans* 6 (60%)	0.52 (±0.28)	0.12–1	0.5	46.86 (±17.11)	31.24–62.48	62.48
*C. glabrata* 2 (20.0%)	0.50 (±0.00)	–	0.5	31.24 (±0.00)	–	31.24
*C. dubliniensis* 2 (20.0%)	0.26 (±0.33)	0.03–0.5	0.5	62.48 (±0.00)	–	62.48
**FCZ-resistant/intermediate**						
*C. albicans* 57 (91.9%)	232.07 (±71.88)	4–256	256	63.02 (±28.61)	31.24–124.95	62.48
*C. glabrata* 4 (6.5%)	24.00 (±9.23)	16–32	16	46.86 (±18.03)	31.24–62.48	62.48
*C. dubliniensis* 1 (1.6%)	256	–	–	124.95	–	–

Among the FCZ-resistant /intermediate strains, the mean MIC for *C. albicans, C. glabrata* and *C. dubliniensis* were 232.07, 24.00 and 256 μg/ml, respectively (Table 2).

The MIC ranges for K21 with *C. albicans* and *C. glabrata* were 31.24–124.95 and 31.24–62.48 μg/ml, respectively with a MIC_50_ of 62.48 μg/ml, compared to a higher MIC of 124.95 μg/ml for *C. dubliniensis* (Table 3).

**Table 3:** Comparison of mean MIC for Fluconazole and K21 using Student’s *t*-test.

*Candida* strains	Mean (SD)		*T*	*df*	*p* (two-tailed)
	Fluconazole (FCZ)	K21			
Susceptible	0.46 (±0.25)	46.86 (±16.46)	8.87	9	0.000
Resistant	219.03 (±86.15)	62.98 (±29.11)	−14.69	61	0.000

The difference between the resistant and susceptible *Candida* clinical isolates when exposed to FCZ and K21 was significantly different (Table 3).

Having established the quantitative action of K21, we proceeded to examine the qualitative changes to *C. albicans* only, using electron microscopy.

### SEM

**Figure 6:**
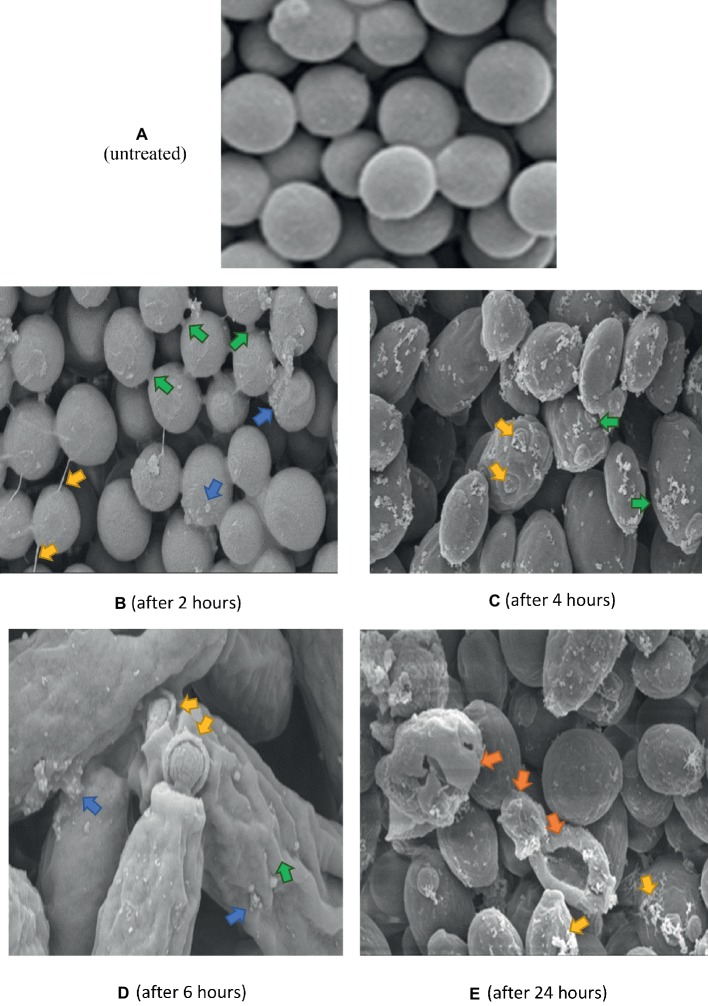
SEM of *C. albicans* (ATCC 90028) treated with K21 after 2, 4, 6 and 24 h. **(A)** untreated cells (Control) show smooth unattached cells except where budding into daughter cells occurred. Magnification = 5.00 kX. After 2 h **(B)**, cells are joined by fine threads (yellow arrows), some showed budding and separation into two cells (green arrows). Others showed small extracellular vesicles (blue arrows). The cells were mostly smooth. Magnification = 5.00 kX. After 4 h **(C)**, cells showed an increase in extracellular vesicles (green arrows) with bud scars not confined to the polar region (yellow arrows). Magnification = 10.00 kX. After 6 h **(D)**, cells were distorted with convoluted surfaces (green arrow), increased extracellular vesicles (blue arrows) and disrupted budding (yellow arrows). Magnification = 20.00 kX. After 24 h **(E)**, cells were severely distorted (orange arrows) with flocculent deposits resembling a biofilm matrix (yellow arrows). Magnification = 10.00 kX.

Untreated *C. albicans* cells (Figure 6A), appeared smooth, with polar buds, and budding into two daughter cells was also observed. After 2 h exposure to K21 (Figure 6B), cells appeared interconnected by thin threads (arrows), and although their surfaces appeared smooth, sparse white vesicles were observed on some cells. This became more pronounced after 4 h (Figure 6C), with the white vesicles increasing significantly with bud scars randomly positioned on convoluted cell surfaces and not defined to the polar region of the cell only (Figure 6C). The distortion of cells became more pronounced after 6 h (Figure 6D), with total lysis of the cell observed after 24 h (Figure 6E).

**Figure 7:**
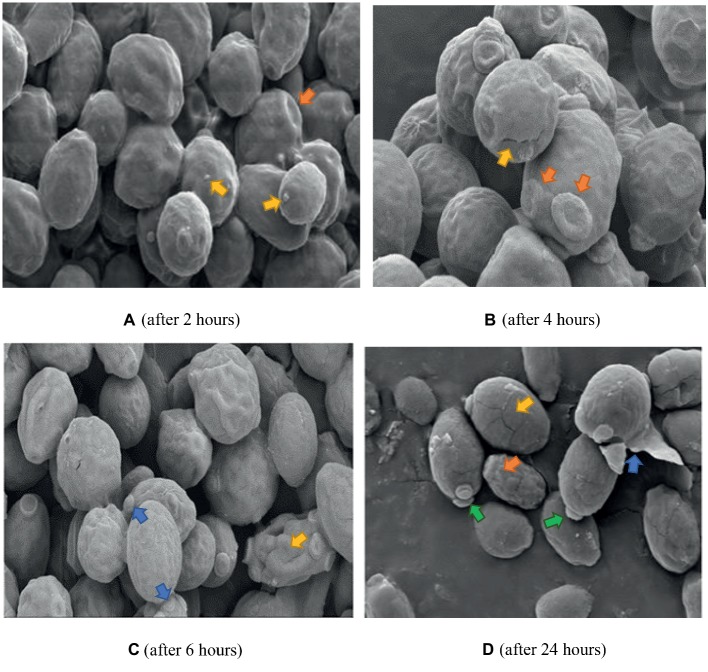
SEM of *C. albicans* (ATCC 90028) treated with FCZ after 2, 4, 6 and 24 h. Untreated cells in previous figure served as the control for Figures 7 and 8 as well. **(A)** Cell surfaces appear shrunken and wrinkled with sparse extracellular vesicles (yellow arrows) and bud scars (orange arrow). Magnification = 5.00 kX. **(B)** Desiccated cells with cracks (yellow arrow), multiple bud scars (orange arrows) and convoluted surfaces. Magnification = 10.00 kX. **(C)** Shrunken desiccated cells with some severely distorted (yellow arrow) and multiple bud scars (blue arrows). Magnification = 5.00 kX. **(D)** Cracked (yellow arrow) and lysed desiccated cells (blue arrow), some totally distorted (orange arrow) and others with polar buds (green arrows). Magnification = 10.00 kX.

**Figure 8:**
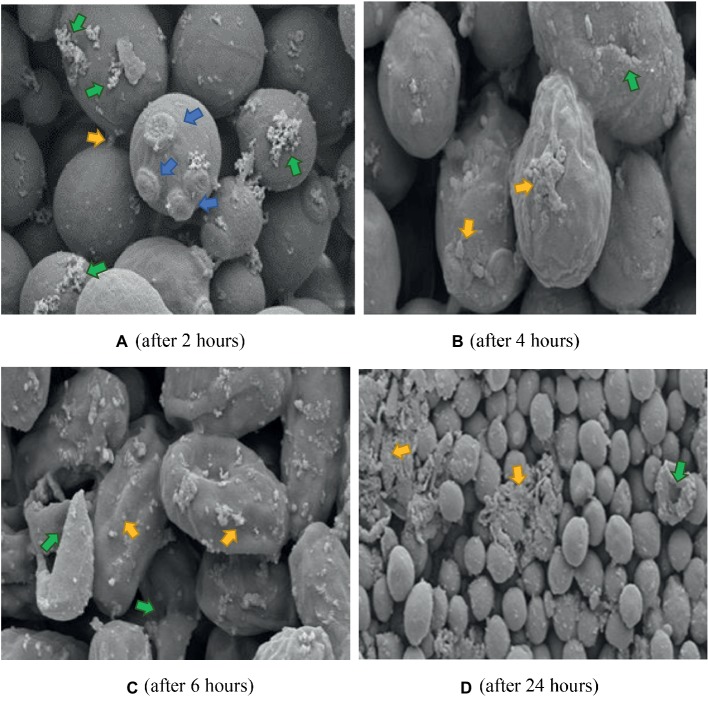
SEM of *C. albicans* (ATCC 90028) treated with K21 + FCZ after 2, 4, 6, and 24 h. **(A)** Cells connected by appendages and appear to be separating (yellow arrow), multiple bud scars are evident, not limited to polar regions of cell (blue arrows), and an increase in flocculent deposits (green arrows). Magnification = 10.00 kX. **(B)** Severely distorted cells with larger extracellular vesicles (yellow arrows) and cracking (green arrow) Magnification = 10.00 kX. **(C)** Totally distorted and disintegrating cells (yellow arrows) with evidence of lytic cell remnants (green arrows). Magnification = 10.00 kX. **(D)** Severely disrupted cells (green arrow), masses of extracellular vesicles and cell remnants (yellow arrows). Magnification = 2.00 kX.

The same control used in Figure 6 applies to Figures 7 and 8 since they were all prepared from the same microdilution plate. Cells treated with FCZ demonstrated shrinkage after treatment for 2 h (Figure 7A), with wrinkled cell surfaces. Bud scars were evident and sparse vesicles were observed on a few cells. The cells appeared cracked and desiccated after 4 h (Figure 7B), with multiple polar scars evident on some cells (arrow). This became more pronounced after 6 h (Figure 7C), where total distortion of cells, with multiple bud scars were observed. After 24 h of exposure to FCZ, cells appeared cracked with broken cell walls and some cellular remnants with further evidence of multiple bud scars and extracellular vesicles (Figure 7D).

The K21 + FCZ combination after 2 h exposure did not show the wrinkled cells when treated with FCZ on its own (Figure 8A). Several bud scars were apparent on the cells along with an increase in the white flocullar material (arrows). After 4 h (Figure 8B), the cells were elongated with several showing larger extracellular vesicles and debris on convoluted cell surfaces. This progressed over 6 h showing severely distorted and lysed cells with extracellular vesicles and deposits of white flocculent material (Figure 8C). After 24 h, masses of cellular remnants were observed along with distorted and lysed cells (Figure 8D) with no evidence of the fine flocculent material described earlier. Several damaged buds not limited to polar regions were evident and the cell surfaces were convoluted and lysed.

### TEM

**Figure 9:**
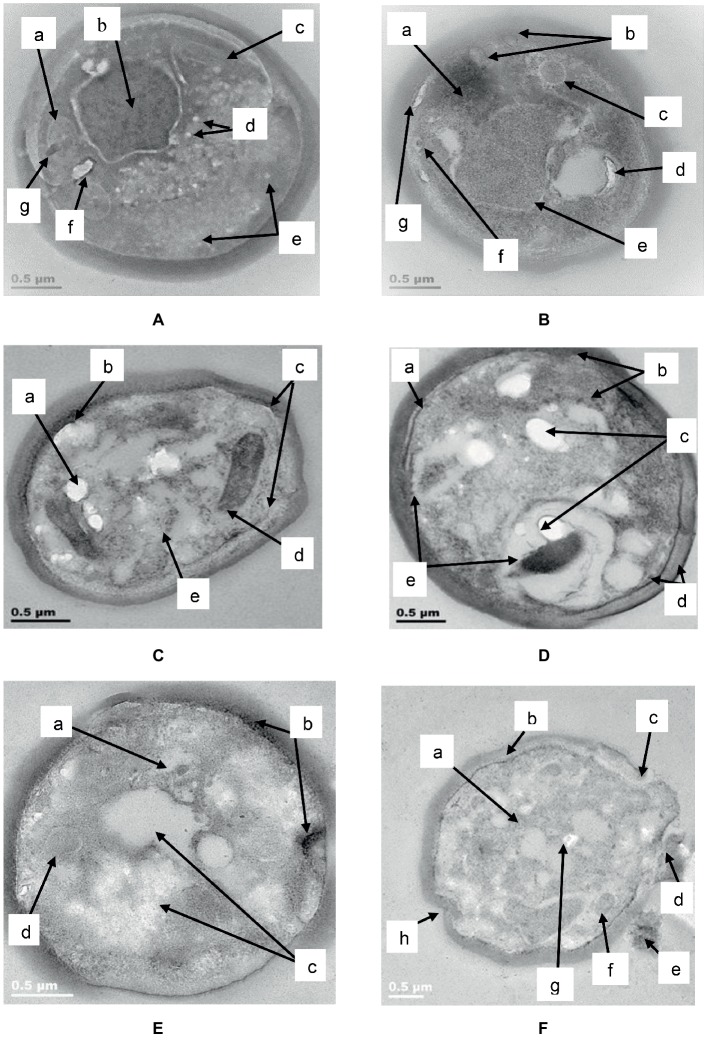
TEM of *C. albicans* (ATCC 90028) treated with K21. In the untreated cell **(A)** the cell wall and attached cell membrane enveloped the cytoplasm of the cell which contained cellular organelles (a), a well-defined nucleus with prominent nucleolus (b), mitochondria (c), tiny intracellular vesicles arranged close to the nucleolus (d), and around the periphery of some parts of the cell membrane (e). A vacuole (f) and an electron dense body were also observed (g). After 2 h **(B)**, electron dense material appeared in an amorphous mass (a), the cell membrane showed “blebs” separating it from the cell wall (b) with intracellular vesicles (c) and vacuoles (d). The nucleus showed shrinkage (e) and a tiny vesicle with an electron dense center (f) could be seen near to the bleb forming along the cell membrane (g). After 4 h **(C)**, the lipid deposits increased (a) and melanin granules were deposited along the periphery of the cell membrane (b). There was thickening of the cell wall and disruption of the cell membrane (c). Vesicular compartments contained electron dense material (d) and other electron dense vesicles were seen inside the cell membrane along with vacuoles (e). After 6 h **(D)**, melanin deposits were seen along the periphery of the cell membrane (a), with several other larger electro dense vesicles observed outside of the cell wall as well as within the cell (b). We observed an increase in lipid deposits (c), melanin deposits (d) chromatin condensation and vacuoles (e). After 24 h **(E)**, condensation of the nucleus (a), melanin deposits along the cell membrane along with changes in the membrane were seen (b), as well as vacuoles (c) and large vesicle forms (d). Another cell **(F)** after 24 h in K21, revealed vacuole formation (a), melanin deposits along the cell membrane (b), separation of the cell wall from the cell membrane where a bleb had formed (c), a vesicle being released (d) and extracellular remnants with electron dense inclusions (e). Other vesicle (f) and lipid forms (g) were also observed, along with invaginations of the cell wall and membrane (h).

TEM analysis of untreated *C. albicans* cells (Figure 9A) showed a distinct cell wall and cell membrane with regular cellular contents (a), a well-defined nucleus with prominent nucleolus (b) and other intracellular organelles such as mitochondria (c). A few white vesicles were observed close to the nucleolus (d) and around the periphery of the cell membrane in some parts (e). A vacuole (f) and an electron dense body (g) were visible to the left of the nucleolus.

After 2 h (Figure 9B) of exposure to K21 (31.24 μg/ml), an amorphous mass of electron dense material was observed (a), the cell wall remained intact, while the cell membrane showed “blebs” along its periphery separating it from the cell wall (b). Formation of intracellular vesicles (c) and vacuoles (d) were apparent. The nucleus showed shrinkage (e), and a tiny vesicle with an electron dense center (f) was observed in proximity to the bleb formation along the cell membrane (g).

After 4 h, (Figure 9C) the lipid deposits increased (a) and several melanin granules were observed along the periphery of the cell membrane, along with thickening of the cell wall (b). Disruption of the cell membrane to initiate bleb formation was evident (c) as well as vesicular compartments containing electron dense material (d). Many of these electron dense particles were scattered throughout the cell along with vacuoles (e).

After 6 h (Figure 9D), evidence of bleb initiation was observed where melanin deposits and other electron dense material appeared along the periphery of the disrupted cell membrane (a) with several other tiny electron dense deposits observed within the cell membrane and extracellularly (b). Lipid deposits increased (c) and melanin deposits were seen along the periphery of both the cell membrane and the cell wall in some parts of the cell (d). Chromatin condensation was evident along with a few vacuoles (e).

Following 24 h of exposure to K21*, C. albicans* showed pronounced shrinkage of the nucleus (a) with melanin deposited along the periphery of the cell membrane especially where blebs were starting to form (b). Vacuoles increased significantly (c), with general destruction of intracellular contents (Figure 9E). Another cell (Figure 9F), showed extensive vacuole formation after 24 h (a), with melanin deposits along the cell membrane (b). The cell wall and cell membrane had separated where blebs had formed (c) and were in the process of being released (d). Some cellular remnants with tiny electron dense material were observed extracellularly (e), with vesicles (f) and lipid forms (g) observed within a highly vacuolated and disorganized cytoplasm, along with invaginations of the cell wall and membrane (h).

## Discussion

Drug overuse and the inappropriate prescription of drugs may result in the mutation of organisms (Read and Woods, 2014), leading to the evolution of resistant microbes and associated complications (Lushniak, 2014). This has serious implications for the treatment of OPC, particularly in South Africa where *Candida* carriage was reported to be higher than in other parts of the world (Patel et al., 2006), with the rate in HIV-positive subjects (83%) being significantly higher (*p* < 0.001) than in HIV-negative subjects (63%).

The present study analyzed the antifungal activity of K21 against nine *Candida* type strains, followed by the testing of randomly selected isolates from HIV infected patients using broth microdilution, a technique considered to be the golden standard for antimicrobial susceptibility references.

Even though K21 was found to be effective at higher concentrations compared to FCZ, in the present study, the action of K21 as an antifungal agent can be embraced. However, to inhibit the growth of the resistant *C. albicans* strains, FCZ exhibited a MIC_50_ of 256 μg/ml. Hence it can be noted that K21 is effective against FCZ-resistant strains.

The time kill study is used to determine the fungicidal or fungistatic action of a drug or a compound and the relationship between the drug concentration and microbial activity over time and although time-consuming and expensive, it is widely used to determine the synergy of drug combinations (Shrestha et al., 2015). In the present study, time kill assays for K21 with *C. albicans* (ATCC 90028 and NCPF 3281), *C. dubliniensis* (NCPF 3949a) and *C. glabrata* (ATCC 26512) were evaluated by the checkerboard method. Although the results gained by both methods were similar, *C. glabrata* (ATCC 26512) demonstrated a synergistic interaction at ½ MIC when K21 and FCZ were combined. The indifferent interaction between K21/FCZ with *C. krusei (*ATCC 2159) and *C. glabrata* (ATCC 26512) may be due to the resistant mechanisms of the strains.

We elected to use only a susceptible *C. albicans* (ATCC 90028) and resistant *C. glabrata* (ATCC 26512) to determine the rate of action of K21, because these are the predominant species responsible for causing widespread fungal infection in both healthy and immunocompromised patients (Leroy et al., 2009), with *C. glabrata* being extremely difficult to treat due to its high resistance to fluconazole. The current study showed that 2 h was required to kill 99.9% of both *C. albicans* and *C. glabrata* at a concentration of 62.48 μg/ml. The ½ MIC and ¼ MIC showed fungistatic activity with a growth peak up to 6 h indicating that K21 can be considered as an alternative to FCZ for the treatment of resistant strains.

The FICI indices showed definite synergy for *C. dubliniensis* (NCPF 3949a), *C. tropicalis* (ATCC 950) and *C. lusitaniae* (ATCC 34449). Low MIC values were observed at ΣFICI for all of the nine type strains. None of the combinations exhibited antagonism in the study. This suggests that K21 acts as a potent antifungal agent when combined with fluconazole.

Electron microscopy revealed the different events which occurred following treatment with K21 and we propose that the extracellular vesicles observed after 2 h were distinctly different from those observed after 24 h. The vesicles observed initially, appear to be an indication that the cells were wanting to survive the effects of the antifungals by protecting themselves by forming a biofilm within which they might survive. We observed the presence of melanin granules in and around the cell membrane after 4 h of exposure to K21 in TEM sections. These were similar to those reported by Walker et al. (2010) after 3 days of exposure to 1 mM DOPA (3,4,dihydroxyphenylalanine-melanin). Melanins are biological compounds thought to represent an amorphous mixture of polymers exported from the fungal cells by means of the fusion of cell and vesicular membranes. Melanins are involved in a range of virulence properties including survival and resistance to antifungal agents (Nosanchuk and Casadevall, 2003). It is not clear whether extracellular vesicles must cross the cell wall to be released by the interaction with other cell wall components (Wolf et al., 2014), whether it originates and is extruded from the cytoplasm and exported extracellularly (Eisenman et al., 2009), or comes about as a result of membrane budding and multivesicular bodies (Rodrigues et al., 2013). The integrity of the cell membrane is vital to the survival of the fungal cell, providing a barrier to environmental stress. Thus many of the metabolic processes of the cell are to be found there. Ergosterol is abundant in fungal cell membranes and plays an important role in regulating permeability and fluidity of the membrane as well as cell division (Hata et al., 2010) and is an important target for most antifungals including Polyenes (Amphotericin B, deoxycholate, Liposomal lipid complex) which bind directly to ergosterol, thereby destroying the integrity of the cell membrane (Kim et al., 2011). Vesicles are reported to be composed of proteins, lipids, polysaccharides, pigments and nucleic acids with release from its cell of origin occurring as a result of the outward budding of the cell membrane (ectosomes) (Minciacchi et al., 2015) or inward budding of the endosomal membrane forming multivesicular bodies which, upon fusion with the cell membrane release exosomes (Rodrigues et al., 2008; Oliveira et al., 2010). Lastly, they can also be referred to as apoptotic bodies released when cell membrane blebbing occurs during apoptosis. Vesicles containing melanin are sometimes rearranged by the alteration of cell wall components (Eisenman et al., 2009) and *C. albicans* has been reported to transport RNA *via* its extracellular vesicles (Peres da Silva et al., 2015).

Controversy exists regarding the nomenclature and size distribution of the different types of EVs (Gould and Raposo, 2013), despite minimal criteria for their classification having been defined (Lötvall et al., 2014) and thus, it is accepted that the term EV be used until specific markers are able to classify them (Abels and Breakefield, 2016). Because of the discrepancies regarding the characterization of the different EVs, interpretation of data involving EVs create limitations in drawing accurate conclusions (Caruso and Poon, 2018).

The morphological changes observed by SEM and TEM in the present study are not unlike those observed in other studies which investigated the effect of other azoles and classes of antifungals. *In vitro* treatment of *C. albicans* with Miconazole (10^−7^ M) showed increases in buds as well as bud scars on several regions of the cell wall indicating that Miconazole interferes with the normal division of the yeast cells causing a single cell to make multiple attempts to divide and survive (De Nollin and Borgers, 1975). Because of the fungistatic activity of the drug, viable cells were not able to divide resulting in the formation of clusters of interconnected cells as observed after exposure for 2 h to both K21 as well as FCZ in the present study. When exposed to the minimal fungicidal dose (10^−6^ M) and fungicidal doses (10^−4^ M) of Miconazole, De Nollin and Borgers (1975) observed smooth cells coated with vesicles and because large amounts of fragmented cell walls were observed, they presumed that the vesicles were cytoplasmic components derived from broken cells. They also observed that cells which appeared normal by SEM, showed necrosis by TEM. TEM showed cytoplasmic membrane material limited to the cell membrane with a variety of intact cell walls. Thus they cautioned that it cannot be taken for granted that a normal shape and size of cell need necessarily indicate viability, particularly since they found that the fungicidal dose brought about lytic changes while the cell wall was unaffected in part of the population. They also observed an accumulation of membranous material at sites of what appeared to be bud formation.

Combination therapies increase efficacy thereby preventing drug resistance. Different approaches have identified effective combinations of natural compounds with marketed drugs (Prasad and Kapoor, 2005; Spitzer et al., 2017). Mady et al. (2018) demonstrated synergistic activity between Miconazole and urea for treating *C. albicans*. Other examples include the use of novel compounds such as APX001A and APX2020 in combination with FCZ with a FICI of 0.37 (Shaw et al., 2018), calcium channel blockers such as amlodipine, nifedipine, bendipine, and flunarizine with FCZ with FICI <0.5 (Liu et al., 2016), licofelone and FCZ (Liu et al., 2017). Studies of the interaction between baicalein (a flavonoid isolated from the roots of *Scutellaria baicalensis*, a Chinese medicinal plant) and FCZ showed that the fungicidal activity of FCZ + baicalein was greater than that of each agent individually, thus demonstrating synergy between the two. SEM revealed that *C. albicans*, when exposed to baicalein alone, produced filamentous forms with flocculent extracellular material when combined with FCZ, suggestive of biofilm formation (Serpa et al., 2012). We observed several features consistent with their findings and others, which may suggest that for the first few h, the activity of K21 appeared to be fungistatic (as indicated by the microdilution and EM observations), followed by fungicidal activity.

Several forms of cell death have been proposed, all of which have been defined by morphological criteria (Kroemer et al., 2009). In this study, we have demonstrated different events leading to cell death. The presence of multiple buds, tiny vesicles and flocculent deposits suggest a desperate attempt at cell survival (autophagy) by budding and formation of a protective biofilm, followed by oncosis (as revealed by melanin deposits along the cell membranes, loss of cell membrane integrity with bleb formation) and finally, apoptosis (demonstrated by cell shrinkage and the production of extracellular apoptotic bodies and cell remnant deposits) (Kroemer et al., 2009; Weerasinghe and Buja, 2012). Previous studies reported apoptosis in *C. albicans* at low fungicidal doses of Amphotericin B and necrosis in high fungicidal doses (Phillips et al., 2003), while Hao et al. (2013) found that Caspofungin had a harmful effect on the cell wall without the cytoplasm being affected. They demonstrated chromatin margination and condensation along the nucleus, concluding that large black dots and blebs from the nucleus are indicative of apoptosis.

Apoptosis, a highly organized process leading to cell death and essential for normal growth and maintenance of cells, has also been demonstrated in *C. albicans* following treatment with purpurin, a neutral red anthraquinone pigment in *Rubia tinctorum* L, also known as madder root (Kang et al., 2010). Apoptosis is usually indicated by dysfunction of mitochondria (enlarged, containing electron dense deposits), which are organelles considered to be the energy powerhouse of the cell and also playing an important role in cellular integrity (Wang and Youle, 2009), along with protein aggregation, chromatin condensation, increase in number and size of lipid droplets, vacuoles and cytoplasmic autolysis (Kang et al., 2010), all of which have been observed in cells treated with K21 in this study.

Other studies using natural products and showing characteristics of apoptosis include Coprisin (peptide from dung beetle) (Lee et al., 2012) and ethanolic crude extracts of *Punica granatum* L (pomegranate) (Anibal et al., 2013).

Since K21 alone or in combination with FCZ has proved its effectiveness against both FCZ- resistant and susceptible *Candida* isolates, it can be considered that the above *in vitro* study renders the evolvement of the novel compound K21 as a possible alternative to fluconazole. Furthermore, a study by Daood et al. (2017), reported the effectiveness of K21 as a dental cavity disinfectant, thus introducing its use in oral health care as an antimicrobial agent, while a cytotoxic study on K21 found no major toxic effects on primary human fibroblasts (Gulve et al., 2016), further indicating the competency in the application of the compound.

This study recognized the phenotypical differences between the type strains and clinical isolates in their antimicrobial susceptibility, thereby confirming the value of investigating clinical isolates along with type strains in the pathogenic potential and antifungal activity of *Candida* species (Silva et al., 2010). Laboratory adapted type strains may undergo micro-evolution during several *in vitro* subcultures since their first isolation, thus not suitably representing current clinical strains when it comes to drug susceptibility (Andes et al., 2004).

In the present study the antifungal activity of K21 against *Candida* type strains and fluconazole-resistant oral *Candida* species showed that K21 inhibited the growth of FCZ-resistant *Candida* at a faster rate, demonstrating a potent feature of the compound, thereby suggesting it be considered for the development of a curative therapy for all forms of fungal diseases, particularly in HIV-associated opportunistic infections.

Limitations of this study include our failure to determine whether a similar *in vitro* efficacy with K21 could be observed with other drug classes besides the azoles and that we limited the electron microscopy to *Candida albicans* only. However, this study has set the stage for future studies to gain a better understanding of the mechanism of the antifungal activity of K21.

The need for the development of innovative antifungal agents with a rapid killing action is critical to prevent the spread of invasive fungal infections and the long-term use of antifungals, thereby reducing or preventing the emergence of resistant *Candida* species. K21, with its fast-acting fungicidal property may create a new chapter in the discovery of new antifungal agents.

## Author Contributions

CA conceptualized and coordinated the study, participated in its design and wrote the final manuscript. CJ performed the antimicrobial investigations, analyzed the data and prepared the first draft of the manuscript. PA characterized the *Candida* isolates and contributed to the editing of the manuscript. BP and DA contributed to the formulation of the protocol and editing of the manuscript. All authors read and approved the final manuscript.

### Conflict of Interest Statement

The authors declare that the research was conducted in the absence of any commercial or financial relationships that could be construed as a potential conflict of interest except for a grant from fiteBac Technology which covered a PhD bursary for CJ. All other authors declare no competing interests.
